# Rare variants at *KCNJ2* are associated with LDL-cholesterol levels in a cross-population study

**DOI:** 10.1038/s41525-024-00417-9

**Published:** 2024-06-28

**Authors:** Niccolò Rossi, Najeeb Syed, Alessia Visconti, Elbay Aliyev, Sarah Berry, Mafalda Bourbon, Tim D. Spector, Pirro G. Hysi, Khalid A. Fakhro, Mario Falchi

**Affiliations:** 1 Department of Twin Research & Genetic Epidemiology, King’s College London, London, UK; 2 Department of Human Genetics, Sidra Medical and Research Center, Doha, Qatar; 3 Center for Biostatistics, Epidemiology and Public Health, Department of Clinical and Biological Sciences, University of Turin, Turin, Italy; 4 Department of Nutritional Sciences, King’s College London, London, UK; 5 Cardiovascular Research Group, Department of Health Promotion and Prevention of non-Communicable Diseases, Instituto Nacional de Saúde Dr. Ricardo Jorge, Lisbon, Portugal; 6 Department of Genetic Medicine, Weill-Cornell Medical College, Doha, Qatar

**Keywords:** Genome-wide association studies, Genetics research

## Abstract

Leveraging whole genome sequencing data of 1751 individuals from the UK and 2587 Qatari subjects, we suggest here an association of rare variants mapping to the sour taste-associated gene *KCNJ2* with reduced low-density lipoprotein cholesterol (LDL-C, *P* = 2.10 × 10^−12^) and with a 22% decreased dietary trans-fat intake. This study identifies a novel candidate rare locus for LDL-C, adding insights into the genetic architecture of a complex trait implicated in cardiovascular disease.

## Introduction

Circulating lipids, and in particular LDL-cholesterol (LDL-C), have been extensively studied due to their role in atherosclerosis pathogenesis. While numerous common genetic loci associated with LDL-C have been described so far^1^, the study of the contribution of rare variants to LDL-C variation remains an under-explored area, which has the potential to unveil novel therapeutic targets and improve risk profiles. Rare variants association studies represent a major challenge of genetic research, as they require sufficiently large sample sizes for a well-powered analysis, and they are prone to replication failure across diverse populations as a consequence of potential heterogeneity of populations in the genetic structure and environmental exposures^2,3^. So far, only a few association studies^4,5^ have been successful in uncovering novel, rare loci associated with LDL-C. Identified associated variants fall in the coding portion of the genome due to the expected strong effect of coding variants on phenotypic variation.

The TwinsUK dataset^6^ consisted of high coverage (30×) WGS data of 1751 UK subjects, including 344 monozygotic and 425 dizygotic twin pairs and 213 singletons (Supplementary Table 1). After quality control, a total of 58,461,543 rare and common single nucleotide variants (SNVs) were identified. We first performed a single-point genome-wide association study (GWAS) for LDL-C on 7,859,673 common (MAF ≥ 1%) SNVs. Results confirmed the absence of population stratification or other confounding biases (*λ*_GC_ = 1.02; Supplementary Fig. 1a). The analysis identified 29 common variants significantly associated with LDL-C at the customary significance threshold of *P* < 5 × 10^−8^. These variants mapped to seven unique loci on chromosome 19: *APOC1, APOC1P1, APOE, BCAM, LDLR, NECTIN2*, and *TOMM40* (Supplementary Fig. 1b, Supplementary Table 3), whose associations with LDL-C were either previously reported or in high linkage disequilibrium (LD; *r*^2^ ≥ 0.8) with variants reported in the literature^7^.

**Fig. 1:**
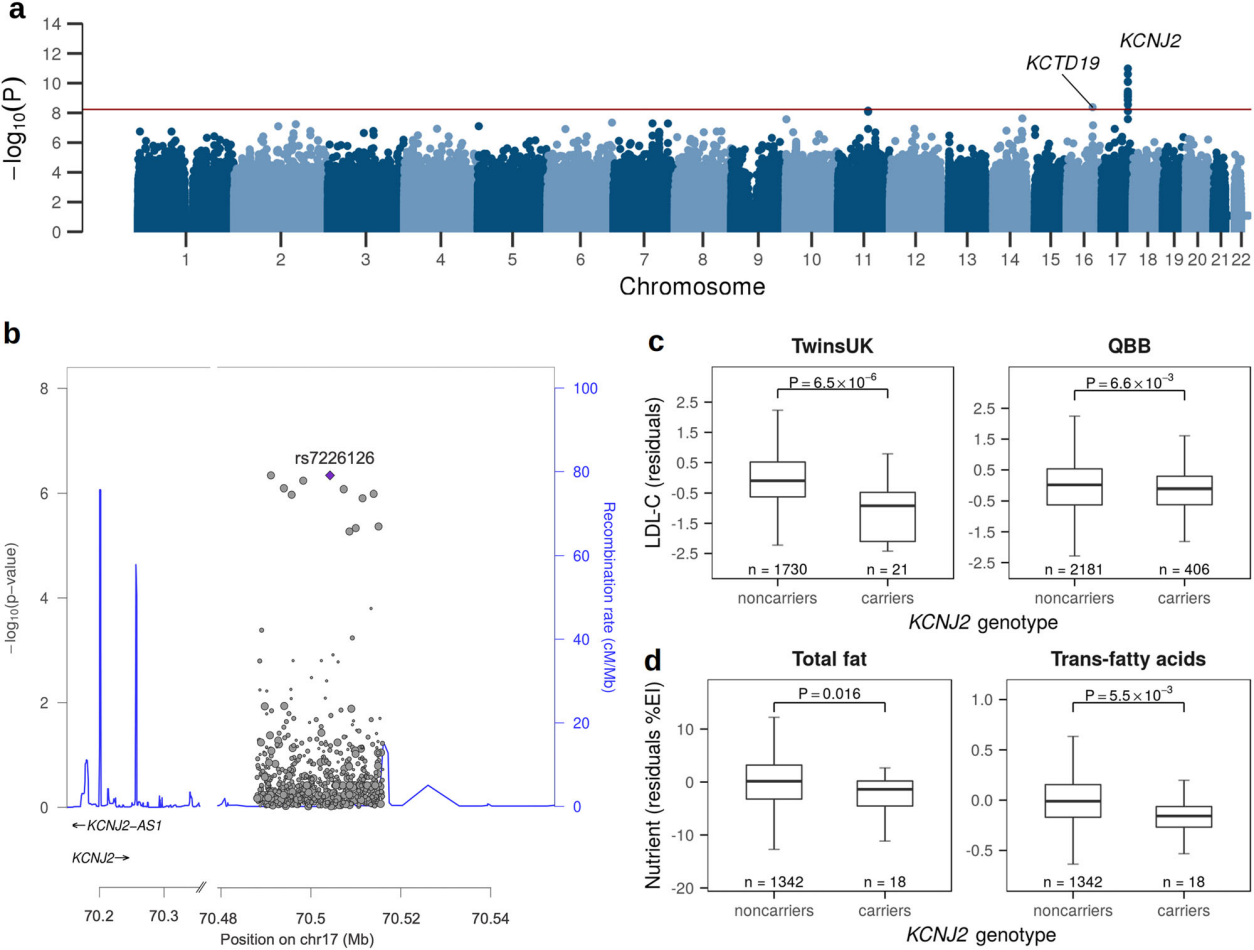
Association of rare variants at *KCNJ2* with LDL-C and fat intake. **a** Manhattan plot of region-based rare (TwinsUK MAF < 1%) variants association for LDL-C in TwinsUK. Each dot corresponds to the p-value for a 4-kb window. Genome-wide significance threshold (*P* < 6 × 10^−9^) is indicated by the red horizontal line. For each significant locus, the closest gene is shown. **b** Regional Manhattan plot of the association with LDL-C on chromosome 17 downstream of *KCNJ2*. Dots represent the combined *P*-value in TwinsUK and QBB at each SNV, with the top SNV rs7226126 shown in purple. **c** Boxplots of the distribution of LDL-C (adjusted for age, sex, and BMI) in TwinsUK and QBB among carriers of any of the rare lead variants for LDL-C at *KCNJ2* compared to non-carriers. **d** Boxplots of the distribution of total fat and trans-fatty acid intake (expressed as a percentage of daily total energy intake, %EI, and adjusted for age and BMI) in TwinsUK among carriers of any of the rare lead variants for LDL-C at *KCNJ2* compared to non-carriers. The boxplots centre lines indicate the median and the limits the 25th and 75th percentile. The whiskers represent either 1.5 times the interquartile range or the maximum/minimum data point if they are within 1.5 times the interquartile range. Reported *P*-values are from Wilcoxon’s tests.

We then carried out a rare variant region-based GWAS for LDL-C, using a SKAT-O method accommodating sample relatedness^8^, interrogating 1,341,836 sliding windows, each of a length of 4000 base-pair, comprising a total of 50,601,870 rare (MAF < 1%) SNVs. We replicated previous rare-variants associations for LDL-C at the Mendelian dyslipidaemia genes *LDLR*, *APOB*, *APOE*, and *PCSK9*^9^ (*P* < 1.27 × 10^−3^), and identified novel candidate associations at 12 windows at a significance threshold for discovery of 6 × 10^−9^ (Supplementary Tables 4, 5). Eleven overlapping windows, spanning 26 kb of chromosome 17 (chr17:70,493,859–70,519,858, genomic coordinates GRCh38; *P*_min_ = 1.03 × 10^−11^), localised ~300 kb downstream of the *KCNJ2* gene, and one 4-kb window (chr16:67,304,097–67,308,096*;* *P* = 4.16 × 10^−9^) within the *KCTD19* gene (Fig. 1a, Supplementary Table 5). A multi-ancestry meta-analysis of lipid levels in 1.65 million individuals identified genome-wide significant associations between common variants at *KCNJ2* (lead SNV = rs9890133, *P* = 6.17 × 10^−10^, MAF_EUR_ = 0.119) and LDL-C^10^. Conditioning on rs9890133 or other common variants located within 500 kb and previously reported in association with lipids, adiposity measures, or heart conditions did not affect the significance of our associations (Supplementary Tables 6, 7).

Replication was conducted in 2587 individuals from the Qatari Biobank (QBB) cohort^11^ for whom WGS data was available. Four out of 11 4-kb windows within the 26-kb region mapping to *KCNJ2* were replicated in QBB at a Bonferroni-adjusted threshold of 7.14 × 10^−3^ (*P*_min_ = 2.72 × 10^−3^), with the highest association from meta-analysis at chr17:70,493,859–70,497,858 (*P*_combined_ = 2.10 × 10^−12^; Supplementary Table 5). The region encompassing the *KCTD19* gene was not replicated (*P* > 0.05).

To dissect the contribution of individual SNVs to the aggregate variants associated with LDL-C, we performed single point association of 92 rare (MAF_TwinsUK_ < 1%) variants included in the chr17:70,493,859–70,519,858 region, and present in both TwinsUK and QBB. Lead SNVs rs7226126 and rs80345346, 13 kb apart and in complete LD in both TwinsUK and QBB, were associated at *P* = 1.71 × 10^−6^ (*β* = −1.19; SE = 0.25; MAF = 0.5%) in TwinsUK and *P* = 9.67 × 10^−3^ in QBB (*β* = −0.14; SE = 0.05; combined *P* = 4.70 × 10^−7^; Fig. 1b), where they showed higher minor allele frequency (MAF ~ 0.07, Supplementary Table 8). Allele frequencies were comparable between females and males in the general population (Supplementary Table 9). Effect allele frequency breakdown by sex and ancestry is reported in Supplementary Table 9. The near 10-fold lower MAF observed in TwinsUK as compared to QBB at rs7226126 (and rs80345346) corresponded to approximately a ten-fold higher phenotypic effect size for LDL-C among carriers. Among carriers of at least one of these variants, average LDL-C (after accounting for sex, age, and BMI) was 0.97 (SD = 0.22) and 0.12 (SD = 0.05) mmol/L lower than non-carriers in TwinsUK and QBB, respectively (Fig. 1c).

The *KCNJ2* gene encodes for an acid-sensitive potassium channel (K_ir_2.1) involved in the sour taste transduction cascade^12^. K_ir_2.1 is inhibited by intracellular acidification resulting from hydrogen ions from acidic food entering sour-sensing taste cells^12^. While *KCNJ2* is relatively ubiquitously expressed and contributes to the resting potassium potential in both sour and non-sour taste cells, the lower expression of *KCNJ2* in sour taste cells makes them more sensitive to intracellular acidification^12^, suggesting that *KCNJ2* expression levels are determinant for sour taste transduction and sensitivity. The region chr17:70,493,859–70,519,858 encompasses expression quantitative trait loci (eQTLs) for *KCNJ2* and its antisense gene *KCNJ2-AS1* (Supplementary Fig. 2), and overlaps a putative transcription factor binding site for *KCNJ2* (chr17:70,503,507–70,503,756; OREG1689846^13^), suggesting that rare variants harboured within this region may affect *KCNJ2* expression and, in turn, sour taste sensitivity. This is supported by previous evidence that sour taste sensitivity is strongly (>50% of the variance) heritable^14^ and that common genetic variants with putative regulatory effects on *KCNJ2* expression (i.e., intronic or 3’UTR variants) are associated with sour food preference^15^. As taste sensitivity can affect food preference and intake^16^, with sour food consumption being itself (34–50% of the variance) heritable^17^, we hypothesised that the association between rare variants at *KCNJ2* and LDL-C could be explained, at least in part, by *KCNJ2* effect on food preference. We tested the association between aggregated rare variants at chr17:70,493,859–70,519,858 and nutrient intake from food frequency questionnaires which were available for 1360 female subjects from TwinsUK (Supplementary Table 10). Multiple macronutrients (i.e., total fat, monounsaturated fatty acids, trans fatty acids, total sugars, and starch) were significantly associated with aggregated rare variants at chr17:70,493,859–70,519,858 at a Bonferroni-adjusted threshold of 5.56 × 10^−3^ (Supplementary Table 11), with total fat intake being the most significant (*P* = 7.54 × 10^−4^).

Particularly, among carriers of the lead rare variant rs7226126 (or rs80345346), a smaller percentage of the daily total energy intake was derived from fat (*β* = −2.82%; SE = 1.28%, *P* = 2.82 × 10^−2^), and a larger percentage from total sugar and starch (*β* = 4.91%, SE = 1.36%, *P* = 2.98 × 10^−4^ and *β* = −3.56%, SE = 1.13%, *P* = 1.67 × 10^−3^, respectively; Supplementary Table 11) compared to non-carriers, corresponding to a relative reduction of 10% total fat intake and, more importantly, 22% trans fatty acids intake (Fig. 1d). While this is consistent with studies reporting that dietary reductions in trans fatty acids and their substitution by carbohydrates may have a beneficial effect on serum lipids^18^, the interaction between sour taste sensitivity and dietary fat intake remains to be elucidated. Interestingly, while a link between oral fat sensitivity, fat intake and obesity has long been established^19^, the existence of a fat-taste perception mechanism independent of other taste qualities is still debated, with a study showing that short-chain fatty acids stimulate a sensation similar to sour^20^. This evidence suggests that individuals with a preference for sour-tasting foods may also favour a diet rich in fat. Notably, the *KCNJ2* synonymous variant rs173135-T, located ~300 kb upstream chr17:70,493,859–70,519,858 and reported for higher sour preference in 44 Canadian families from the Guelph Family Health Study^15^, has been associated with total energy density of diet (i.e., calories per food weight units) in the same cohort^15^, with fat having the highest energy density compared to other nutrients. The same allele has been reported for increased serum levels of the ketone body 3-hydroxybutyrate in 2482 Dutch genetic isolates from the Erasmus Rucphen Family (ERF) study^21^, with ketone bodies being produced on high-fat, low-carbohydrate (i.e., ketogenic) diets. Also, a genetic variation within chr17:70,493,859-70,519,858 has been associated with adiposity measures^22^. Interestingly, an association between increased BMI and lower sour taste sensitivity has been observed^23^.

In summary, here we report the association results of genome sequencing data of a total of 4338 individuals from two populations. We suggest that a burden of rare variants mapping to the sour taste-associated gene *KCNJ2* contributes to lowered LDL-C levels despite population heterogeneity in terms of allele frequency and effect size. We propose that this finding may be due to the KCNJ2 effect on food preference, as rare variant carriers from the UK had reduced dietary fat intake compared to non-carriers. The present study, while warranting further investigation, suggests *KCNJ2* as a novel candidate rare-variant locus for LDL-C.

## Methods

### Discovery cohort

Study subjects were 1751 twins, mainly females (97%; Supplementary Table 1), belonging to the TwinsUK cohort, a UK national register of volunteer adult twins unselected for any diseases or traits^6^. St. Thomas’ Hospital Research Ethics Committee approved the study, and all twins provided informed written consent.

### LDL-cholesterol data

Subjects from TwinsUK had data for longitudinal plasma biochemical indices of HDL-cholesterol (HDL-C), triglycerides (TG) and total cholesterol (TC). Fasting plasma levels were measured using an analysing device (Cobas Fara; Roche Diagnostics, Lewes, UK). TC, HDL-C, and TG were determined by a colorimetric enzymatic method^24^. A median of four longitudinal measures was available per subject (the maximum number of measures was 8). The median duration of follow-up was 5 years (interquartile range: 2.87–7.51). Longitudinal data on medication usage indicated that 292 subjects were using cholesterol-lowering drugs at the time of lipid measurement, and their effect was taken into account by dividing the measured total cholesterol by 0.8, as previously suggested^25^. LDL-C values were estimated based on adjusted TC, HDL-C, and TG levels, according to the Friedewald equation^26^. Longitudinal LDL-C data were averaged, inverse-normal transformed, and adjusted for sex, age, and BMI by linear regression analysis (using the *lm* function as implemented in the *stats* R package, v. 3.4.2). No outliers (individuals with any trait value further away than four standard deviations from the dataset mean) were detected.

### Whole-genome sequencing and quality control

Whole genome sequencing was carried out at Human Longevity, Inc. (HLI). Details on sample preparation, library preparation, clustering and sequencing have been reported elsewhere^27^. Reads were mapped to the UCSC GRCh37 human reference genome, and variants were called using Illumina Isaac Analysis Software^28^ (v. 2.5.26.13). SNVs calls that did not pass all Isaac’s filters (see Isaac Whole Genome Sequencing user guide for details: https://support.illumina.com/content/dam/illumina-support/documents/documentation/software_documentation/basespace/isaac-wgs-user-guide-15050954b.pdf) were discarded as low-quality calls. Multiallelic sites were removed, and missing genotypes were assumed to be reference homozygous calls. We further removed SNV with calls rate <95% and/or Hardy–Weinberg equilibrium (HWE) deviation *P* < 1 × 10^−9^, as calculated with PLINK^29^ (v. 1.9). We finally removed variants mapping to the sex chromosomes, resulting in 58,461,543 biallelic SNVs.

To investigate population structure, we used PLINK to carry out a principal component analysis (PCA) on the genomic data. Here, we used all the common high-quality variants (i.e., missingness across variants = 0 and MAF > 5%) pruned with PLINK (option: --indep-pairwise) using the following parameters: window size of 50-kb, shift of 5 variants, and *r*^2^ threshold of 0.05. No significant population stratification was observed (Supplementary Fig. 3).

### Single-point association testing

SNVs with MAF ≥ 1% were tested for association with sex, age, and BMI-adjusted plasma LDL-C levels using a linear mixed model as implemented in GEMMA^30^ (v. 0.97), which uses the matrix of expected genetic sharing to model the non-independence of the twin data. Manhattan and Q–Q plots were generated using the *ggplot2* R package. Significant SNVs at conventional genome-wide significance threshold (*P* < 5 × 10^−8^) were annotated with Ensembl VEP web interface (v. 94) using the NCBI Reference Sequence Database (release 2015-01).

### Identification of known LDL-C loci

We interrogated the NHGRI-EBI GWAS catalog^7^ (v. 1.0, release: 2019-01-11; association *P* < 5 × 10^−8^) to identify overlap between SNVs identified in our study (or in high linkage disequilibrium with them; *r*^2^ ≥ 0.8) and previously reported associations for LDL-C levels. Linkage disequilibrium statistics were calculated with PLINK.

### Region-based associations testing

Region-based testing was carried out using MONSTER^8^ (v. 1.3), which uses a generalised version of the SKAT-O method for non-independent samples to perform robust region-based rare-variant association testing. Briefly, this programme uses a mixed effects model that accounts for covariates and additive polygenic effects, accounting for relatedness among individuals and adaptively estimating the correlation structure of variant effects to maximise the statistical power. Coefficients of the relationship were assumed to be 1 for monozygotic twins and 0.5 for dizygotic twins. We assessed associations by collapsing rare (MAF < 1%) variants within fixed-size sliding windows. Specifically, 1,341,836 sliding windows of 4 kb (overlapping by 2 kb) were generated beginning at position 1 bp for each chromosome, as described previously^31–33^). The median number of SNVs per window was 48 (interquartile range: 39–65). We opted for a stringent genome-wide significance threshold of 6 × 10^−9^, following suggestions from previous simulation studies for WGS-based analytic strategies combining individual common variants testing and aggregated rare variants tests using the sliding window approach^34^).

### Region-based conditional analysis

We used secondary conditional analyses to assess the independence of signals of association from previously known GWAS signals arising from common variation near the chr17:70,493,859–70,519,858 and chr16:67,304,097–67,308,096 regions. Specifically, we retrieved from the NHGRI-EBI GWAS catalog^7^ (v. 1.0, release: 2019-01-11) any reported SNV associated with blood lipids, adiposity or cardiovascular risk within 500 kb on either side of the studied regions. Then, we fitted a new region-based regression model for LDL-C as implemented in MONSTER, including the reported SNVs as a covariate.

### Replication cohort

The Qatar Genome Programme (QGP) is a national population-based initiative aiming at combining a comprehensive analysis of Qatari genomes with the extensive phenotypic data collected at the QBB^11^. All subjects are enroled in the Qatar Biobank via informed consent. The Qatar Biobank study was approved by the Institutional Review Board from the Hamad Medical Corporation Ethics Committee (IRB protocol E/2017/RES-ACC-0032/0002). Whole genome sequencing was performed on 2935 blood samples using Illumina X10 Sequencing machine, with a 150-base paired-end single-index-read format with an average coverage of 30×. Raw WGS data were converted from the native BCL format to paired-end FASTQ format using bcl2fastq (v. 2.16). The quality of the raw data was assessed using fastqc (http://www.bioinformatics.babraham.ac.uk/projects/fastqc/). Sequence reads were aligned to the human reference genome version GRCh37 using bwakit (v. 7.12) (https://github.com/lh3/bwa/tree/master/bwakit). Variant calling was performed using GATK haplotype caller^35^ (v. 3.3). SNVs with call rate <95% or Hardy–Weinberg equilibrium (HWE) deviation *P* < 1 × 10^−9^ were discarded from the analysis. We leveraged genotypes at 48 ancestry-informative SNVs to differentiate the three major Qatari subpopulations (Bedouin Arabs, Persians, and East Africans) in the QBB cohort, as previously described^36^. We excluded from the analysis individuals of African ancestry due to the small sample size (*n* = 63), as well as 78 individuals, mainly (*n* = 75, 96%) admixed individuals, separated from the main cohort core based on PC2 value (PC2 < −0.03). Coefficients of the relationship between subjects were modelled using the kinship matrix between all individuals, as evaluated by PLINK (v. 1.9), to account for both familial and more distant relatedness.

LDL-C was measured from blood samples at the Hamad Medical Centre Laboratory, Doha, inverse-normal transformed and adjusted for sex, age and BMI. Outliers (measurements further away than four standard deviations from the dataset mean) were removed from subsequent analyses, which were ultimately conducted on 2587 individuals. The phenotypic details of the study subjects are summarised in Supplementary Table 2.

Food frequency data was not available for the QBB cohort.

### Meta-analysis

Meta-analysis of window-based association results was carried out by combining *P*-values across TwinsUK and QBB using Fisher’s combined probability test, as implemented in *metap* R package (v. 1.1, *sumlog* function). A Bonferroni-adjusted threshold of 7.14 × 10^−3^ was obtained by dividing a conventional *α*-threshold of 0.05 by the number of non-overlapping 4-kb windows tested for replication (i.e., six windows mapping to *KCNJ2* and one window to *KCTD19*).

Individual SNVs values were meta-analysed using a sample-size weighted *Z*-score-based analysis, as implemented in METAL^37^ (release 2011-03-25), and their sex- and ancestry-specific allele frequencies were downloaded from the gnomAD browser^38^ (v. 3.1.2). The regional association plot was generated using LocusZoom^39^. Reported eQTLs for *KCNJ2* and *KCNJ2-AS1* (Ensembl gene identifiers ENSG00000123700.4 and ENSG00000267365.1, respectively) were retrieved from GTEx^40^ (v. 8) and eQTLGen^41^ (phase I) portals.

### Food frequency data

Overall, 1360 female subjects from the TwinsUK cohort with WGS data available completed a 131-item validated food frequency questionnaire^42^ (FFQ) between 1993 and 2015 and had BMI data available within ±1 year of completing the FFQ. These included 345 dizygotic pairs, 218 monozygotic pairs, and 234 singletons (Supplementary Table 10). Details on quality control, subject exclusion criteria and methods for nutrient determination from FFQ data can be found elsewhere^43^. Briefly, study participants reported food intake frequencies for the past year of average serving sizes for 131 foods and beverages on a 9-point scale (ranging from never or less than once per month to 6+ times per day). The 131 food items were aggregated into food groups, defined by similarity in nutrient content and culinary use, resulting in 54 different nutrients. We considered 12 macro-nutrients for association testing, namely: the four major classes (alcohol, protein, total fat, and total carbohydrate) and eight subclasses of macronutrients (starch, total sugar, fibre, saturated fatty acids, monounsaturated fatty acids, polyunsaturated fatty acids, trans fatty acids and cholesterol), based on their relevance to LDL-C metabolism^44^. Two or more longitudinal measures were available for 53% of the study subjects (the maximum number of measures was 4). The median duration of follow-up was 9 years (interquartile range: 3.57–10.22 years). When longitudinal data were available, nutrients calculated at each timepoint were averaged. Outliers (values further away than four standard deviations from the nutrient mean) were removed.

### Association between *KCNJ2* and nutrients

We tested the association of aggregated rare variants at *KCNJ2* with nutrient intake, adjusted for daily total energy intake, age, and BMI, using MONSTER. Coefficients of relationship were assumed to be 1 for monozygotic twins, and 0.5 for dizygotic twins. To correct for multiple comparisons, we used Li’s method^45^ to estimate the effective number of independent tests in the nutrient dataset. The derived *P*-value threshold for statistical significance was 0.05/9 = 5.56 × 10^−3^.

Subsequently, we used linear mixed model regression analysis (*lmer* function from *lme4* R package, v. 1.1-21) to test for association between lead rare variants at *KCNJ2* and selected nutrients, expressed as percentages of daily total energy intake, modelling family structure as random effect, and including age and BMI as covariates. Average nutrient intakes in the TwinsUK cohort (Supplementary Table 10) fell within the interquartile range of the UK adult population^46^.

### Ethical approval

The TwinsUK study was approved by the National Research Ethics Service London-Westminster, the St. Thomas’ Hospital Research Ethics Committee (EC04/015 and 07/H0802/84). The Qatar Biobank study was approved by the Institutional Review Board from the Hamad Medical Corporation Ethics Committee (IRB protocol E/2017/RES-ACC-0032/0002). Written informed consent was obtained from all participants. All research therefore carried out in accordance with the ethical standards laid down in the 1964 Declaration of Helsinki and its later amendments.

### Supplementary information

Supplementary Material

## Data Availability

Data on TwinsUK twin participants are available to bona fide researchers under managed access due to governance and ethical constraints. Raw data should be requested via our website (http://twinsuk.ac.uk/resources-for-researchers/access-our-data/) and requests are reviewed by the TwinsUK Resource Executive Committee (TREC) regularly. QQB data should be requested by filling out the access application at www.qatarbiobank.org.qa, to be submitted to the research access office accessofficeqbb@qf.org.qa. Requests are reviewed and approved by QBB IRB and Access Committee.

## Code availability

Analyses were carried out using open-source published pieces of software which are referenced in the manuscript.
