# Supplementary material for: Rare variants at KCNJ2 are associated with LDL-cholesterol levels in a cross-population study
Source: NPJ Genom Med. 2024 Jun 28;9:36. doi: 10.1038/s41525-024-00417-9 (PMC11213907; doi:10.1038/s41525-024-00417-9)
Supplement: Supplementary file 1 — Supplementary Material [file 41525_2024_417_MOESM1_ESM.pdf]

Supplementary Material for:

Rare variants at KCNJ2 are associated with LDL-cholesterol levels in a cross-population study

Niccolò Rossi[1], Najeeb Syed[2], Alessia Visconti[1,3], Elbay Aliyev[2], Sarah Berry[4], Mafalda Bourbon[5], Tim D. Spector[1], Pirro G. Hysi[1], Khalid A. Fakhro[2,6*], and Mario Falchi[1*]

[1] Department of Twin Research & Genetics Epidemiology, King's College London, London, UK

[2] Department of Human Genetics, Sidra Medical and Research Center, Doha, Qatar

[3] Center for Biostatistics, Epidemiology and Public Health, Department of Clinical and Biological Sciences, University of Turin, Turin, Italy

[4] Department of Nutritional Sciences, King's College London, London, UK

[5] Grupo de Investigação Cardiovascular, Unidade de I&D, Departamento de Promoção da Saúde e Doenças Crónicas, Instituto Nacional de Saúde Dr Ricardo Jorge, Portugal

[6] Department of Genetic Medicine, Weill-Cornell Medical College, Doha, Qatar

Supplementary Tables

Supplementary Table 1. Descriptive statistics of TwinsUK study participants. For each continuous variable, we report mean, standard deviation, and 1st,3rd interquartile range (in brackets).

	**All** (N=1,751)	**Females** (N=1,695)	**Males** (N=56)
**Monozygotic twins**	688	676	12
**Dizygotic twins**	850	808	42
**Singletons**	213	211	2
**Age**	59.07±7.8 [53.83-64.43]	59.03±7.84 [53.53-64.43]	60.17±6.39 [57.43-64.41]
**BMI (kg/m^2)**	26.22±4.56 [23.05-28.49]	26.17±4.6 [22.99-28.46]	27.67±2.96 [25.84-29.46]
**LDL-C (mmol/L)**	3.55±0.95 [2.88-4.09]	3.54±0.95 [2.88-4.09]	3.58±0.94 [3.08-4.01]

Supplementary Table 2. Descriptive statistics of QBB study participants. For each continuous variable, we report mean, standard deviation, and 1st,3rd interquartile range (in brackets).

	**All** (N=2,587)	**Females** (N=1,318)	**Males** (N=1,269)
**Age**	39.26±12.14 [29-48]	39.55±12.67 [29-49]	38.97±11.56 [30-46]
**BMI (kg/m^2)**	28.8±5.66 [24.9-32.3]	29.24±6.2 [24.9-33.27]	28.33±5 [24.9-31.2]
**LDL-C (mmol/L)**	2.98±0.87 [2.35-3.52]	2.88±0.8 [2.23-3.3]	3.09±0.91 [2.48-3.66]

Supplementary Table 3. Results of single-point association study in TwinsUK. For each mapped gene, we report the strongest associated SNV (genomic coordinates GRCh38) along with its minor allele frequency (MAF), effect size (β) and standard error (SE), association p-value (P), and the SNV within loci previously identified by LDL-C GWASs (NHGRI-EBI GWAS catalog [DOI: 10.1093/nar/gkt1229]; association $P<5x10^{-8}$) either coincident or in LD ($r^2>0.8$) with our lead SNV (NHGRI-EBI).

Gene	SNV	Variant	MAF (%)	β	SE	P	NHGRI-EBI
APOC1	rs445925	19:44912383:G:A	11.9	-0.46	0.06	$3.45x10^{-16}$	rs445925
APOC1P1	rs141622900	19:44923535:G:A	6.1	-0.68	0.08	$1.09x10^{-18}$	rs141622900
APOE	rs7412	19:44908822:C:T	9.0	-0.58	0.06	$4.80x10^{-21}$	rs7412
BCAM	rs118147862	19:44816374:G:A	5.3	-0.58	0.08	$2.46x10^{-21}$	rs118147862
LDLR	rs10412048	19:11083273:A:G	12.1	-0.31	0.05	$1.78x10^{-8}$	rs6511720
NECTIN2	rs41290120	19:44879418:G:A	5.6	-0.64	0.08	$1.21x10^{-15}$	rs41290120
TOMM40	rs1160983	19:44893972:G:A	3.2	-0.69	0.11	$9.65x10^{-11}$	rs1160983

Supplementary Table 4. **Region-based rare variant associations at known Mendelian dyslipidaemia genes.** For each gene identified as part of previously published WGS-based rare variant association study for LDL-C [DOI: 10.1038/s41467-018-05747-8], we report the strongest associated 4-kb window located within 50 kb around the gene (genomic coordinates: GRCh38), the number of rare SNVs included in the tested region (N), and the relative association p-value (P).

Coordinates	Gene	N	P
1:54988327-54992326	*PCSK9*	41	$4.56x10^{-4}$
2:20989128-20993127	*APOB*	45	$1.12x10^{-5}$
19:11105324-11109323	*LDLR*	74	$2.09x10^{-4}$
19:44876743-44880742	*APOE*	68	$1.27x10^{-3}$

Supplementary Table 5. Region-based rare variant associations. For each window (genomic coordinates: GRCh38), we report the closest gene and its distance from the associated window (Distance), the number of rare SNVs included in the tested region in the TwinsUK cohort (N) and the relative association p-value ($P_{TwinsUK}$), the association p-value for aggregated rare variants in the QBB cohort (P_{QBB}), and the combined p-value for aggregated rare variants in TwinsuK and QBB (P_{Fisher}).

**Coordinates**	**Gene**	**Distance**	**N**	$P_{TwinsUK}$	P_{QBB}	P_{Fisher}
16:67304097-67308096	*KCTD19*	0	34	4.16×10^{-9}	>0.05	3.98×10^{-8}
17:70493859-70497858	*KCNJ2*	313,806	45	1.03×10^{-11}	6.52×10^{-3}	2.10×10^{-12}
17:70495859-70499858	*KCNJ2*	315,806	34	5.94×10^{-10}	1.06×10^{-2}	1.69×10^{-10}
17:70497859-70501858	*KCNJ2*	317,806	38	3.59×10^{-10}	6.88×10^{-3}	6.85×10^{-11}
17:70499859-70503858	*KCNJ2*	319,806	37	8.31×10^{-11}	1.09×10^{-2}	2.60×10^{-11}
17:70501859-70505858	*KCNJ2*	321,806	38	7.81×10^{-11}	9.02×10^{-3}	2.04×10^{-11}
17:70505859-70509858	*KCNJ2*	325,806	33	2.46×10^{-11}	1.28×10^{-2}	9.38×10^{-12}
17:70507859-70511858	*KCNJ2*	327,806	41	7.61×10^{-10}	2.72×10^{-3}	5.78×10^{-11}
17:70509859-70513858	*KCNJ2*	329,806	55	5.04×10^{-10}	5.04×10^{-3}	7.04×10^{-11}
17:70511859-70515858	*KCNJ2*	331,806	62	1.35×10^{-9}	2.31×10^{-2}	7.86×10^{-10}
17:70513859-70517858	*KCNJ2*	333,806	50	8.11×10^{-10}	2.26×10^{-2}	4.71×10^{-10}
17:70515859-70519858	*KCNJ2*	335,806	44	1.29×10^{-9}	2.05×10^{-2}	6.71×10^{-10}

Supplementary Table 6. Results of the conditional analysis at chr17:70493859-70519858. Conditional rare-variants region-based associations for LDL-C were evaluated with MONSTER [DOI: 10.1002/gepi.21775]. We included as covariate any SNV located within 500 kb either side of the region and previously identified by GWAS (NHGRI-EBI GWAS catalog [DOI: 10.1093/nar/gkt1229] v1.0, release: 2019-01-11) for adiposity or cardiovascular risk at $P<5x10^{-8}$, with the exception of the suggestive association between SNV rs16975985 and LDL-C ($P=5.00x10^{-6}$). For each conditioning SNV (genomic coordinates: GRCh38), we report its position (Chr:BP), the SNV ID and the risk allele (SNV), its frequency (AF), the reported mapped gene, the association statistics from the original GWAS study (P_{SNV}: GWAS association p-value; ES: effect size; 95% CI: 95% confidence interval; Trait: associated trait), and the conditional p-value for LDL-C (P_{cond}; unconditioned $P=3.58x10^{-10}$). * WHR: waist-hip ratio; PA: physical activity interaction; HC: hip circumference. † odds ratio. ‡ β coefficients.

Chr:BP	SNV	AF	Gene	P_{SNV}	ES	95% CI	Trait*	P_{cond}
17:70172864	rs9890133-G	0.12	*KCNJ2*	$6.17x10^{-10}$	+0.02 U‡	[0.01-0.02]	LDL-C	$3.67x10^{-10}$
17:70207405	rs236586-G	0.47	*KCNJ2*	$6.00x10^{-11}$	+0.64 U‡	[0.44-0.84]	QT interval	$3.49x10^{-10}$
17:70329727	rs10775360-T	0.29	*KCNJ2*	$1.00x10^{-12}$	-0.76 U‡	[0.54-0.98]	QT interval	$4.81x10^{-10}$
17:70341044	rs7219869-G	0.44	*KCNJ2, CASC17*	$1.00x10^{-10}$	1.05†	[1.03-1.06]	atrial fibrillation	$9.33x10^{-10}$
17:70347398	rs312750-A	0.47	*KCNJ16, KCNJ2-AS1*	$3.00x10^{-8}$	-0.01 U‡	-	BMI	$7.47x10^{-10}$
17:70351197	rs3844438-T	0.48	*KCNJ2, CASC17*	$3.00x10^{-9}$	1.05†	[1.03-1.06]	atrial fibrillation	$6.11x10^{-10}$
17:70409853	rs984877-A	0.33	*KCNJ2*	$1.00x10^{-8}$	+0.03 U‡	[0.02-0.04]	BMI-adjusted WHR, PA	$1.98x10^{-10}$
17:70425662	rs1396517-T	0.50	*KCNJ2*	$3.00x10^{-10}$	-0.03 U‡	[0.02-0.04]	BMI-adjusted HC	$4.47x10^{-10}$
17:70434852	rs1396515-C	0.52	*KCNJ2*	$2.00x10^{-25}$	-0.98 U‡	[0.8-1.16]	QT interval	$1.21x10^{-9}$
17:70457204	rs8066985-A	0.50	*KCNJ2*	$1.00x10^{-9}$	+0.03 U‡	[0.02-0.04]	BMI-adjusted WHR	$1.45x10^{-9}$
17:70498851	rs17779747-T	0.35	*KCNJ2*	$6.00x10^{-12}$	-1.02 ms‡	[0.53-1.51]	QT interval	$6.53x10^{-11}$
17:70512851	rs173269-A	0.34	*KCNJ2*	$3.00x10^{-8}$	+0.03 U‡	[0.02-0.04]	BMI-adjusted WHR, PA	$7.03x10^{-10}$
17:70525720	rs7219669-T	0.38	*KCNJ2*	$6.00x10^{-14}$	-1.7 ms‡	-	TPE interval	$3.29x10^{-10}$
17:70564648	rs17763769-A	0.15	*KCNJ2*	$5.00x10^{-11}$	+0.89 U‡	[0.62-1.16]	QT interval	$4.39x10^{-10}$
17:70637924	rs16975985-G	0.08	*KCNJ2*	$5.00x10^{-6}$	+0.02 mg/dL‡	-	LDL-C	$4.36x10^{-10}$
17:70648048	rs17718586-?	0.10	*AC007423.1, AC005771.1*	$2.00x10^{-8}$	1.53†	[1.32-1.78]	sudden cardiac arrest	$4.79x10^{-10}$

Supplementary Table 7. Results of the conditional analysis at chr16:67304097-67308096. Conditional rare-variants region-based associations for LDL-C were evaluated with MONSTER [DOI: 10.1002/gepi.21775]. We included as covariate any SNV located within 500 kb either side of the region and previously identified by GWAS (NHGRI-EBI GWAS catalog [DOI: 10.1093/nar/gkt1229] v. 1.0, release: 2019-01-11) for either lipids or adiposity at $P<5x10^{-8}$. For each conditioning SNV (genomic coordinates: GRCh38), we report its position (Chr:BP), the SNV ID and the risk allele (SNV), its frequency (AF), the reported mapped gene, the association statistics from the original GWAS study (P_{SNV}: GWAS association p-value; β: beta coefficient; 95% CI: 95% confidence interval; Trait: associated trait), and the conditional p-value for LDL-C (P_{cond}; unconditioned $P=4.16x10^{-9}$). * WC: waist circumference; PA: physical activity interaction; WHR: waist-hip ratio.

**Chr:BP**	**SNV**	**AF**	**Gene**	**P_{SNV}**	**β**	**CI**	**Trait ***	**P_{cond}**
16:67191598	rs3729639-T	0.44	*EXOC3L1-E2F4*	$2.00x10^{-11}$	+0.09 U	[0.064-0.116]	HDL-C	$1.04x10^{-8}$
16:67219616	rs143264468-C	0.001	*LRRC29*	$2.00x10^{-9}$	-1.16 mg/dl	[0.78-1.53]	HDL-C	$4.38x10^{-9}$
16:67301066	rs16957304-A	0.93	*KCTD19*	$5.00x10^{-9}$	+0.06 U	[0.04-0.08]	BMI-adjusted WC	$4.81x10^{-9}$
16:67346647	rs7184253-?	0.88	*LRRC36*	$2.00x10^{-8}$	-	-	BMI-adjusted WC, PA	$3.54x10^{-9}$
16:67357715	rs8055190-C	0.87	*LRRC36*	$4.00x10^{-9}$	+0.05 cm	[0.035-0.071]	BMI-adjusted WC, PA	$3.54x10^{-9}$
16:67424348	rs6499129-A	0.43	*ZDHHC1-HSD11B2*	$5.00x10^{-8}$	+0.05 U	[0.025-0.069]	BMI-adjusted WHR	$6.24x10^{-9}$
16:67542133	rs183208454-T	$7.61x10^{-4}$	*RIPOR1, AC027682.2*	$5.00x10^{-9}$	-1.31 mg/dl	[0.87-1.75]	HDL-C	$4.38x10^{-9}$
16:67656785	rs141279119-G	$7.64x10^{-4}$	*CARMIL2*	$7.00x10^{-9}$	-1.34 mg/dl	[0.89-1.8]	HDL-C	$4.38x10^{-9}$
16:67777687	rs73591976-C	-	*RANBP10*	$1.00x10^{-45}$	-0.078 U	-	HDL-C	$4.08x10^{-9}$

Supplementary Table 8. Replication in QBB of single point rare variant associations at *KCNJ2*. For each SNV falling within chr17:70493859-70519858 and associating in the TwinsUK cohort at $P<1x10^{-5}$, we report the genomic coordinates (GRCh38), the effect allele (EA), and, for both cohorts: the minor allele frequency (MAF), the effect size (β), standard error (SE), the association p-value (P), as well as the combined meta-analysis p-value (P_{MA}), as calculated with METAL [DOI: 10.1093/bioinformatics/btq340], using a weighted Z-score method based on sample size, p-value, and direction of effect in each study.

			**TwinsUK**				**QBB**				
**SNV**	**Chr:BP**	**EA**	**MAF (%)**	**β**	**SE**	**P**	**MAF (%)**	**β**	**SE**	**P**	**P_{MA}**
rs80345346	17:70494966	A	0.5	-1.19	0.25	$1.71x10^{-6}$	7.6	-0.14	0.05	$9.67x10^{-3}$	$4.70x10^{-7}$
rs193113493	17:70497870	A	0.6	-1.15	0.24	$1.53x10^{-6}$	8.3	-0.12	0.05	$1.51x10^{-2}$	$8.21x10^{-7}$
rs59648993	17:70499567	T	0.5	-1.19	0.25	$1.71x10^{-6}$	7.9	-0.12	0.05	$1.75x10^{-2}$	$1.09x10^{-6}$
rs79588467	17:70502151	T	0.5	-1.19	0.25	$1.71x10^{-6}$	7.4	-0.13	0.05	$1.15x10^{-2}$	$5.95x10^{-7}$
rs7226126	17:70508114	A	0.5	-1.19	0.25	$1.71x10^{-6}$	7.6	-0.14	0.05	$9.67x10^{-3}$	$4.70x10^{-7}$
rs7223315	17:70511163	A	0.5	-1.19	0.25	$1.71x10^{-6}$	8.1	-0.12	0.05	$1.48x10^{-2}$	$8.58x10^{-7}$
rs77720061	17:70513832	C	0.5	-1.13	0.25	$7.57x10^{-6}$	8.2	-0.11	0.05	$2.49x10^{-2}$	$4.74x10^{-6}$
rs76481340	17:70515351	T	0.5	-1.19	0.25	$1.71x10^{-6}$	8.2	-0.12	0.05	$1.95x10^{-2}$	$1.27x10^{-6}$
rs16975870	17:70517809	A	0.5	-1.19	0.25	$1.71x10^{-6}$	7.7	-0.12	0.05	$1.71x10^{-2}$	$1.05x10^{-6}$

Supplementary Table 9. Minor allele frequencies of replicated single point rare variants at *KCNJ2* stratified by sex and ethnicity according to gnomAD (v3.1.2). Genomic coordinates (Chr:BP) of the effect allele (EA) are reported in GRCh38 assembly. Allele frequency is expressed as a percentage. AA: African American; MID: Middle Eastern; AJ: Ashkenazi jewish; ADA: Admixed American; SAS: South Asian; EUR: European; EAS: East Asian.

SNV	Chr:BP	EA	Total	Male	Female	AA	MID	AJ	ADA	SAS	EUR (non-Finnish)	EAS	EUR (Finnish)	Amish
rs80345346	17:70494966	A	2.67	2.59	2.75	7.66	5.38	2.71	1.34	1.33	0.66	0.06	0.04	0.00
rs193113493	17:70497870	A	3.80	3.70	3.90	11.40	6.65	3.14	1.79	1.64	0.73	0.06	0.04	0.00
rs59648993	17:70499567	T	1.26	1.19	1.32	2.68	5.70	2.67	0.61	1.64	0.69	0.04	0.04	0.00
rs79588467	17:70502151	T	1.21	1.15	1.27	2.66	5.38	2.65	0.56	1.33	0.65	0.06	0.04	0.00
rs7226126	17:70508114	A	2.69	2.61	2.77	7.70	5.38	2.71	1.34	1.30	0.67	0.06	0.04	0.00
rs7223315	17:70511163	A	2.77	2.68	2.85	7.85	5.41	2.71	1.41	1.61	0.71	0.06	0.04	0.00
rs77720061	17:70513832	C	2.75	2.70	2.80	7.75	6.05	3.15	1.42	1.62	0.71	0.04	0.04	0.00
rs76481340	17:70515351	T	2.61	2.52	2.71	7.30	6.33	2.71	0.71	1.58	0.71	0.06	0.04	0.00
rs16975870	17:70517809	A	2.57	2.54	2.59	7.23	4.78	3.12	1.34	1.32	0.67	0.06	0.04	0.00

Supplementary Table 10. Descriptive statistics of 1,360 female participants from the TwinsUK study included in the nutrient intake analysis. Nutrients are reported here as percentages of the daily total energy intake. The table reports mean, standard deviations (SD), and 1st,3rd interquartile range [IQR]. DZ: dizygotic; MZ: monozygotic.

	**All subjects** [N=1,360]		**DZ twin** [N=690]		**MZ twin** [N=436]	
	**mean±SD**	**IQR**	**mean±SD**	**IQR**	**mean±SD**	**IQR**
**Age**	54.41±9.47	47.76-61.18	53.43±9.16	47.64-60.04	56.46±9.37	51.09-62.5
**BMI**	25.61±4.40	22.66-27.67	25.58±4.46	22.55-27.69	25.53±4.27	22.71-27.6
**Daily total energy intake (kcal[kJ])**	1,952±511 [8,166±2,137]	1,587-2,271 [6,641-9,502]	1,957±516 [8,189±2,161]	1,583-2,282 [6,625-9,548]	1,943±501 [8,128±2,098]	1,595-2,244 [6,673-9,388]
**Carbohydrate**	51.43±5.35	48.02-54.94	51.46±5.41	48.22-54.99	51.55±5.40	48.06-55.04
**Sugar**	25.90±5.37	22.35-29.25	25.88±5.35	22.34-29.30	26.20±5.53	22.97-29.42
**Starch**	24.88±4.57	21.94-27.45	24.98±4.47	22.08-27.48	24.70±4.67	21.68-27.47
**Total fat**	31.45±5.04	28.14-34.67	31.45±5.14	27.99-34.79	31.28±4.96	28.24-34.45
**Saturated fatty acid**	11.50±2.55	9.80-13.09	11.47±2.55	9.75-13.09	11.47±2.56	9.90-12.80
**Polyunsaturated fatty acid**	7.10±1.63	6.02-8.05	7.11±1.63	6.09-8.04	7.04±1.68	5.79-8.12
**Monounsaturated fatty acid**	10.34±1.97	9.01-11.61	10.30±1.99	8.97-11.61	10.34±1.98	9.07-11.56
**Trans fatty acid**	0.77±0.26	0.60-0.92	0.76±0.25	0.59-0.91	0.79±0.28	0.62-0.95
**Protein**	17.13±2.41	15.45-18.63	17.08±2.38	15.43-18.59	17.17±2.49	15.39-18.68

Supplementary Table 11. Association results for rare variants at chr17:70493859-70519858 and nutrients intake in TwinsUK. Significant associations at Bonferroni corrected threshold of $P<5.56x10^{-3}$ between aggregated rare variants at chr17:70493859-70519858 and nutrient intake, adjusting for daily total energy intake, age, and BMI. We report the results using aggregated variants ($P_{MONSTER}$). We further report the results of the association of nutrient intake using a mixed linear model, expressed as percentages of the daily total energy intake, between carriers and non-carriers of at least one of the nine rare lead variants associated with LDL-C at chr17:70493859-70519858: effect size β (% EI) as a percentage of the daily total energy intake, standard error (SE) and p-value (P_{SNVs}). Family structure was modelled as a random effect, while age and BMI were included as covariates in the model.

Nutrient	N	$P_{MONSTER}$	β (% EI)	SE	P_{SNVs}
Total fat	1,358	$7.54x10^{-4}$	-2.82	1.28	$2.82x10^{-2}$
Monounsaturated fatty acid	1,358	$2.13x10^{-3}$	-0.99	0.50	$4.89x10^{-2}$
Sugars	1,359	$2.60x10^{-3}$	4.91	1.36	$2.98x10^{-4}$
Trans fatty acid	1,359	$3.46x10^{-3}$	-0.16	0.07	$2.09x10^{-2}$
Starch	1,359	$5.33x10^{-3}$	-3.56	1.13	$1.67x10^{-3}$

Supplementary Figures

a

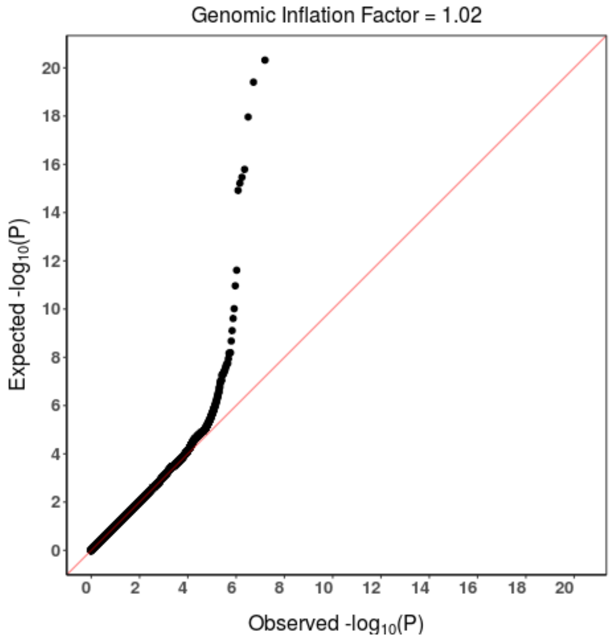

b

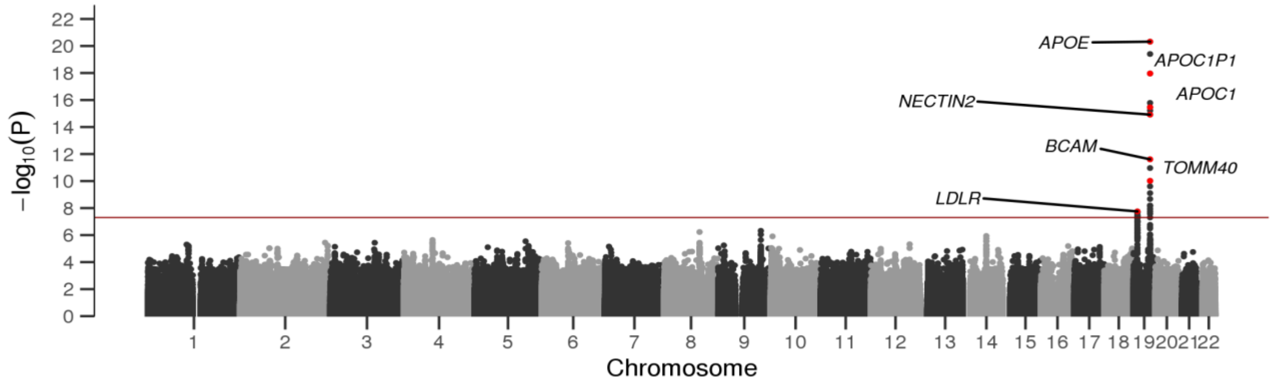

Supplementary Figure 1. Quantile-Quantile and Manhattan plot for the single point GWAS with LDL-C in TwinsUK. (**a**) Single point GWAS Quantile-Quantile plot in TwinsUK. (**b**) Manhattan plots of the associations with LDL-C in TwinsUK. Significant SNVs (MAF≥1%, $P<5x10^{-8}$) were mapped to the closest gene, according to RefSeq annotation. The top-associated SNV for each annotated gene is highlighted in red. Genome-wide significance threshold ($P<5x10^{-8}$) is indicated by the red horizontal line.

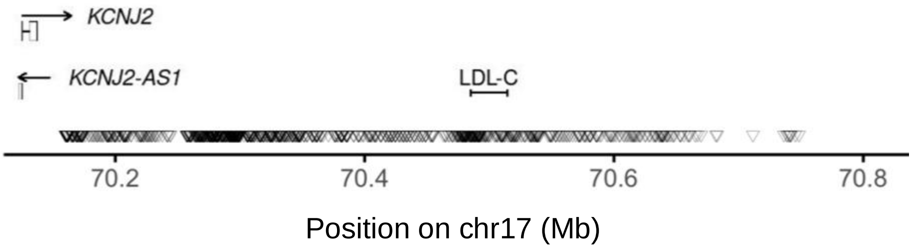

Supplementary Figure 2. Expression quantitative trait loci for *KCNJ2* and *KCNJ2-AS1* reported near chr17:70493859-70519858. Triangles along the x-axis indicate the position of eQTLs affecting *KCNJ2* and *KCNJ2-AS1* expression, as reported in GTEx [DOI: 10.1038/ng.2653] and in the eQTLGen catalogue [DOI: 10.1038/s41588-021-00913-z]. Genomic locations (GRCh38) of the *KCNJ2* and *KCNJ2-AS1* genes, and of the 26-kb region where rare variants associated with LDL-C in our study are shown in the figure.

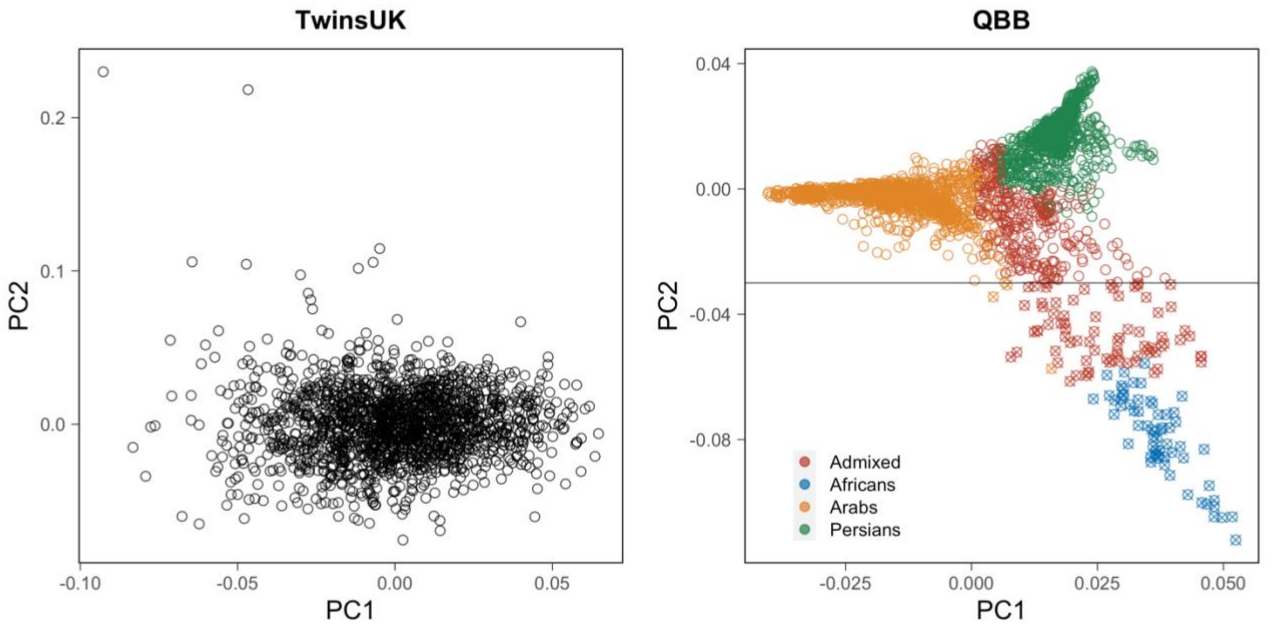

Supplementary Figure 3. PCA plot. Scatter plot of the first two principal components assessed on genome-wide genetic data from TwinsUK (left panel) and ancestry-informative SNPs from QBB (right panel). Within QBB, Africans (n=63), as well 78 admixed individuals separated from the main cohort core were discarded from the analyses.
